# Population imaging cerebellar growth for personalized neuroscience

**DOI:** 10.1038/s41467-024-46545-9

**Published:** 2024-03-18

**Authors:** Zi-Xuan Zhou, Xi-Nian Zuo

**Affiliations:** 1 State Key Laboratory of Cognitive Neuroscience and Learning, Beijing Normal University, No 19 Xinjiekouwai Street, Haidian District, 100875 Beijing, China; 2 Developmental Population Neuroscience Research Center, IDG/McGovern Institute for Brain Research, Beijing Normal University, No 19 Xinjiekouwai Street, Haidian District, 100875 Beijing, China; 3 National Basic Science Data Center, No 04 Zhongguancun South 4th Street, Haidian District, 100190 Beijing, China

**Keywords:** Cognitive neuroscience, Magnetic resonance imaging

## Abstract

Growth chart studies of the human cerebellum, which is increasingly recognized as pivotal for cognitive development, are rare. Gaiser and colleagues utilized population-level neuroimaging to unveil cerebellar growth charts from childhood to adolescence, offering insights into brain development.

The cerebellum contains the majority of neurons in the human brain and is massively connected with most cortical and subcortical areas. Abundant evidence from the past few decades has significantly expanded our understanding of the cerebellum, highlighting its contributions to cognitive functions beyond motor control. Furthermore, despite its prolonged development, vulnerability to disruption, and association with various neurodevelopmental disorders, research on human cerebellar growth in children and adolescents remains limited. Writing in *Nature Communications*, Gaiser and colleagues^1^ leveraged population-level magnetic resonance imaging (MRI) data from the Generation R study^2^ to investigate the morphological growth of the human cerebellum from 6 to 17 years of age with unprecedented scale and detail.

To accurately characterize the general patterns and individual variabilities of cerebellar growth at the population level, Gaiser and colleagues analyzed cerebellar morphometric measurements from MRI scans using the so-called normative modeling approach^3^, constructing normative growth models for both anatomical and functional subregions of the cerebellum, i.e., cerebellar growth charts. Similar to commonly used height or weight growth charts in pediatrics, the normative modeling approach establishes models for the trajectories of population distributions within a transparent statistical framework. Such models enable the derivation of centile scores or normative deviations for corresponding measurements at the individual level (Fig. 1a). While normative modeling has been increasingly adopted to chart normative models of human brain development^4,5^, the cerebellum has rarely been addressed in previous studies. Gaiser and colleagues’ new study fills the gap in brain growth charts for the human cerebellum, providing a valuable resource for studying cerebellar development and its genetic and environmental underpinnings. Furthermore, by investigating the subregional growth rates across the cerebellum, they reported anterior-posterior growth trends, offering insights into an integrative picture of human cerebellar and cerebral development. Finally, they demonstrated the use of the growth charts in detecting cerebellar abnormalities, underlining their potential for personalized neuroscience. Herein, we further discuss the implications of these advances.

**Fig. 1:**
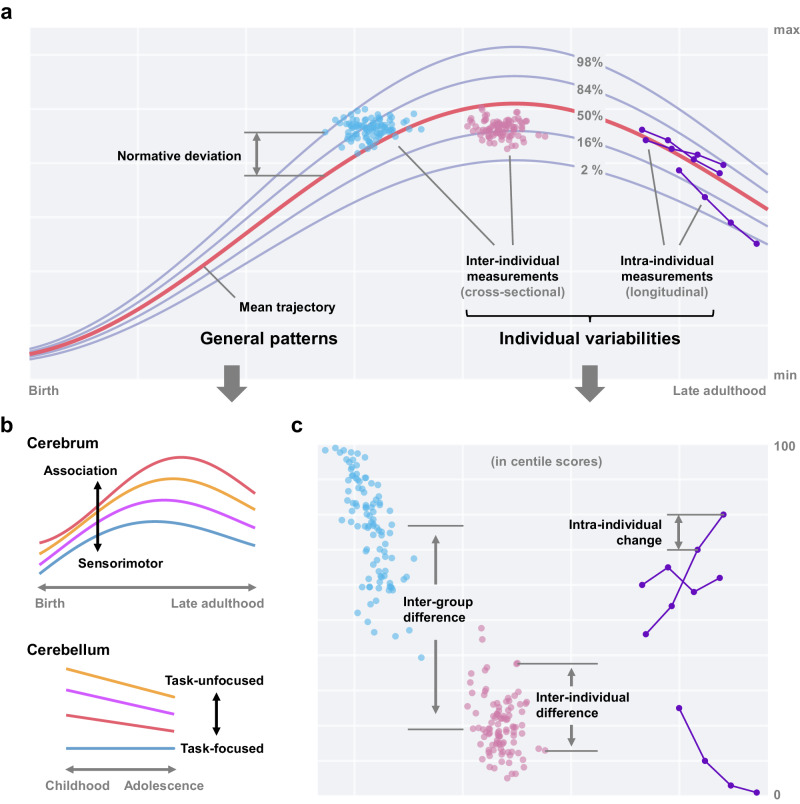
The potential of charting normative models for human brain development. Given a reliable measurement of the human brain, its growth can be charted via normative models of brain development across different life stages (i.e., brain charts) by leveraging large-scale, representative samples. These charts (**a**) offer key insights into general patterns of brain development (**b**) and effectively condense information on individual variabilities for deriving individual-level centile scores or normative deviations (**c**). Brain charts are expected to serve as a better lens for exploring the genetic and environmental underpinnings of population heterogeneity, offering valuable insights and benchmarks for broader neuroscience research, and eventually becoming valid tools in personal healthcare.

## Integrating general principles of human brain growth

With the advent of large-scale cohort resources, human neuroscience has demonstrated the intricate links between inter-individual differences in brain and behavior^6,7^. These findings underline the necessity of exploring the genetic and environmental factors underlying individual variabilities under the guidance of general principles of neurodevelopment. However, current research on human brain development disproportionately emphasizes the cerebral cortex, often overlooking the cerebellum. This neglect hinders a deeper understanding of how the cerebellum contributes to cognitive functions and its association with various neurodevelopmental disorders, not to mention exploring the cerebellum-cerebrum interactions and their cognitive significance during the developmental process. Gaiser and colleagues’ observations on cerebellar growth patterns provide informative cues for integrating general principles of human cerebellar and cerebral development.

Based on a recently proposed functional parcellation of the human cerebellum^8^, Gaiser and colleagues investigated the growth trajectories of gray and white matter density in each functional subregion from childhood to adolescence. Consistent with principles of cerebral development, most cerebellar subregions showed a decrease in gray matter density and an increase in white matter density during this age range. Furthermore, they extracted the standardized age-related coefficients of cerebellar growth from childhood to adolescence for each functional subregion and correlated these growth coefficients with the anterior-posterior spatial positions of the subregions according to their centroids. They detected significant correlations between the cerebellar growth coefficients of gray or white matter density and the anterior-posterior positions of the subregions for both females and males, providing new evidence for the anterior-posterior growth gradient of the cerebellum^9^. Specifically, during this age range, the developmental changes in the motor areas located anteriorly in the cerebellum were significantly less than those in the posterior cognitive areas, suggesting that the cognitive areas in the cerebellum may mature later than the motor areas. This echoes the potential intrinsic developmental gradient from sensorimotor to association cortices in the human cerebral cortex^10^.

Previously, analyses of resting-state functional networks have revealed two functional gradients that effectively characterize the intrinsic cerebral cortical organization^11^, including the sensorimotor-association gradient and the visual-somatomotor gradient (the principal and secondary gradients in young adults, respectively). The changes in cortical gradient patterns over different life stages have been shown in subsequent studies^12^, indicating that the intrinsic developmental sequence of the human cerebral cortex may follow the gradient from sensorimotor to association cortices^10^. Functional gradients for the intrinsic cerebellar organization have also been delineated in young adults with similar approaches^13^, although their relevance to development is rarely explored. By visualizing the distribution of growth coefficients for different functional subregions along the first two cerebellar functional gradients, Gaiser and colleagues observed that the inter-regional differences in the growth coefficients appear to primarily follow the secondary functional gradient from task-unfocused to task-focused processing (Fig. 1b). This suggests that human cerebellar regions involved in task-focused processing (e.g., the frontoparietal network) undergo a longer developmental process than those involved in task-unfocused processing (e.g., motor areas and the default mode network). These observations on cerebellar development align with findings in the cerebral cortex that the frontoparietal network matures later than the sensorimotor cortices but contrast with the longer developmental process of the default mode network. The inconsistency could be resolved by the interactions between the frontoparietal and default mode networks during development^14^. To sum up, Gaiser and colleagues’ findings shed new light on unifying current knowledge about cerebellar and cerebral growth patterns for a holistic picture of human brain development.

## Charting individual variation for personalized neuroscience

Beyond examining the general patterns of cerebellar growth at the population level, Gaiser and colleagues further illustrated the utility of the established cerebellar normative models for benchmarking individual variabilities across different ages/sexes, thereby precisely detecting cerebellar abnormalities at the individual level. Specifically, normative models capture higher-order statistics on sample distributions beyond the mean trajectories. With this prior knowledge on sample distributions, normative models can serve as benchmarks for deriving individual-level centile scores or normative deviations, benefiting downstream analyses or new small-scale studies, which is impossible for models focusing solely on mean statistics.

To fully realize the promise of establishing benchmarks through normative modeling for not only general principles but also individual variabilities, it is imperative to first obtain representative, reliable, and valid measurements of individual variabilities^15^. Gaiser and colleagues have put considerable efforts into approaching this promise. They used a large-scale population-based longitudinal sample and carefully assessed the sample’s representativeness and dropout patterns. They employed advanced cerebellar imaging segmentation tools, including a multi-atlas-based anatomical segmentation algorithm adaptable to different age groups, to improve the modeling of individual variabilities in cerebellar morphology, and visually inspected each MRI scan to ensure segmentation quality. To mitigate the gap between cerebellar morphological anatomy and functional activity patterns, they compared cerebellar growth charts under both anatomical and functional segmentation and observed complementary patterns. To investigate the validity of the established cerebellar growth charts, they evaluated the normative deviations of the cerebellum in a subpopulation of children with autistic traits within the cohort. By revealing the heterogeneity of cerebellar morphology in the subpopulation, they reconciled previously inconsistent findings on the cerebellar anatomy in autism spectrum disorder, demonstrating the unique value of using these growth charts to assess cerebellar normative deviations and understand their neuropathological implications. Furthermore, Gaiser and colleagues also mentioned the prospects of utilizing these charts to benchmark intra-individual longitudinal changes, i.e., quantifying deviations of intra-individual longitudinal trajectories relative to population trends (Fig. 1c). Future studies that quantify intra-individual cerebellar growth with these charts will further validate their potential for personalized applications.

## Concluding remarks

In summary, Gaiser and colleagues leveraged recently available population-level MRI data on human brain development to study the morphological growth of the cerebellum in youth. The study marks an essential step in advancing cerebellar neuroscience through the lens of normative modeling and opens new avenues for exploring cerebellar development in the field of neuroimaging. On one hand, their findings on the cerebellar growth gradient suggest potential coordinated and interactive principles between the human cerebellum and cerebrum during the developmental process. These findings not only highlight the importance of considering both cerebellar and cerebral growth in future explorations but also provide solid evidence for the potential rich rewards of studying cerebellar development. On the other hand, they charted and released, for the first time, normative growth charts of the human cerebellum from childhood to adolescence. These charts provide reliable prior knowledge on both inter-individual and intra-individual variabilities in cerebellar morphology during this age range, promising to serve as a basic resource for personalized cerebellar neuroscience research.
